# Development and Validation of a Generative Artificial Intelligence-Based Pipeline for Automated Clinical Data Extraction From Electronic Health Records: Technical Implementation Study

**DOI:** 10.2196/70708

**Published:** 2026-01-06

**Authors:** Marvin N Carlisle, William A Pace, Andrew W Liu, Robert Krumm, Janet E Cowan, Peter R Carroll, Matthew R Cooperberg, Anobel Y Odisho

**Affiliations:** 1 Department of Urology, University of California, San Francisco, 550 16th Street, Box 1695, San Francisco, CA, 94158, United States, 1 5109126645; 2 Chan Medical School, University of Massachusetts, Worcester, MA, United States; 3 Helen Diller Family Comprehensive Cancer Center, University of California, San Francisco, San Francisco, CA, United States; 4 Department of Epidemiology and Biostatistics, School of Medicine, University of California, San Francisco, San Francisco, CA, United States; 5 Department of Medicine, Division of Clinical Informatics and Transformation, School of Medicine, University of California, San Francisco, San Francisco, CA, United States

**Keywords:** generative artificial intelligence, artificial intelligence large language model, GPT-4, chatbot, pattern analysis, prostate cancer, kidney cancer

## Abstract

**Background:**

The manual abstraction of unstructured clinical data is often necessary for granular clinical outcomes research but is time consuming and can be of variable quality. Large language models (LLMs) show promise in medical data extraction yet integrating them into research workflows remains challenging and poorly described.

**Objective:**

This study aimed to develop and integrate an LLM-based system for automated data extraction from unstructured electronic health record (EHR) text reports within an established clinical outcomes database.

**Methods:**

We implemented a generative artificial intelligence pipeline (UODBLLM) utilizing a flexible language model interface that supports various LLM implementations, including Health Insurance Portability and Accountability Act-compliant cloud services and local open-source models. We used extensible markup language (XML)-structured prompts and integrated using an open database connectivity interface to generate structured data from clinical documentation in the EHR. We evaluated the UODBLLM’s performance on the completion rate, processing time, and extraction capabilities across multiple clinical data elements, including quantitative measurements, categorical assessments, and anatomical descriptions, using sample magnetic resonance imaging (MRI) reports as test cases. System reliability was tested across multiple batches to assess scalability and consistency.

**Results:**

Piloted against MRI reports, UODBLLM processed 1800 clinical documents with a 100% completion rate and an average processing time of 8.90 seconds per report. The token utilization averaged 2692 tokens per report, with an input-to-output ratio of approximately 13:2, resulting in a processing cost of US $0.009 per report. UODBLLM had consistent performance across 18 batches of 100 reports each and completed all processing in 4.45 hours. From each report, UODBLLM extracted 16 structured clinical elements, including prostate volume, prostate-specific antigen values, Prostate Imaging Reporting and Data System scores, clinical staging, and anatomical assessments. All extracted data were automatically validated against predefined schemas and stored in standardized JSON format.

**Conclusions:**

We demonstrated the successful integration of an LLM-based extraction system within an existing clinical outcomes database, achieving rapid, comprehensive data extraction at minimal cost. UODBLLM provides a scalable, efficient solution for automating clinical data extraction while maintaining protected health information security. This approach could significantly accelerate research timelines and expand feasible clinical studies, particularly for large-scale database projects.

## Introduction

### Background

Electronic health record (EHR) systems contain extensive health data, but much of it is in unstructured notes such as radiology and pathology reports, making it hard to access for large-scale research. Granular clinical outcomes research often requires laborious manual chart review. The automation of this process requires significant investment, and algorithm performance varies with report parameters and automation type [1,2]. Previous attempts to automate this process have tried natural language processing on prostate cancer pathology reports, reporting a weighted F_1_ score and accuracy as high as 0.97% and 93%, respectively [3].

Large language models (LLMs) represent a new opportunity for addressing this problem. LLMs are generative artificial intelligence programs capable of drafting human-like responses to specific queries. In oncological contexts, LLM applications can create medical notes, aggregate imaging findings, extract operative note data, and identify presenting symptoms [4-7]. Previous studies analyzing the overall data extraction capabilities have found accuracies ranging from 63.9% to 100% in retrieving data elements [5,8-13]. Specifically, several LLM models have also been developed to extract medical information from text, including early-stage LLM trained on medical encyclopedias and radiology datasets to read annotated radiology reports (71.6% accuracy) and inferring cancer disease response based on computed tomography reports (89% accuracy) [14,15]. Some of these groups also implemented or hypothesized implementing their systems into medical research pipelines for expediting data extraction [3,8]. Another group applied a customized, open-source LLM trained on medical data to read magnetic resonance imaging (MRI) reports with a sensitivity of 96% and specificity of 99%. In terms of data extraction, generative pre-trained transformer (GPT)-4 has been shown to extract hepatocellular carcinoma data from MRI reports with an overall accuracy of 93.4% [16]. LLMs have also proven to be flexible and frequently outperform traditional automated models, suggesting that powerful LLMs might be ready to support research endeavors via the extraction of unstructured data [5,8,17]. Implementing LLMs into practical, applicable tools remains challenging, and some private organizations have attempted to improve clinical data extraction through EHR integration [18]. Despite this, most efforts, such as the American Urological Association Quality Registry, remain dependent on manual data management, partially due to difficulty integrating new tools into existing workflows. While some larger institutions have begun implementing automated data extraction pipelines, traditional methods of data extraction require considerable technical expertise and resources to initiate, making these methods inaccessible for most institutions.

The University of California, San Francisco (UCSF) Department of Urology maintains the Urologic Outcomes Database (UODB) for prostate, bladder, and renal cancers [19]. The UODB is an SQL-based clinical data research database that holds structured manually abstracted clinical data for patients treated at the UCSF, including 7000 patients with prostate cancer over 20 years. Due to limited manual abstraction capacity and increasing patient volume, clinical events and data entry often lag. Previous in-house attempts to automate this process using traditional natural language processing solutions proved to be time-consuming to develop and maintain [1-3,20]. The aim of this study was to demonstrate a practical use of LLMs in academic clinical research by describing the successful implementation of a secure, baseline, institutional version of GPT-4 within the UODB to quickly and easily extract unstructured data and effectively reduce manual labor in gathering data from medical reports.

### Related Work

Previous studies by our group have utilized UCSF’s Versa, an internal, secure, Health Insurance Portability and Accountability Act (HIPAA)-compliant deployment of OpenAI’s GPT models (OpenAI Inc.) that includes an application programming interface (API) for query automation [17,21]. We demonstrated that systems based on the Versa GPT-4 API can accurately extract structured data from real-world clinical reports. In one study involving 424 prostate MRI reports, our pipeline, using zero-shot prompting, achieved an overall median field-level accuracy of 98.1% (IQR 96.3%‐99.2%), with key elements such as prostate-specific antigen density (98.3%), extracapsular extension (97.4%), and TNM staging (98.1%) [21]. In a separate effort with 228 prostate MRI reports, the approach achieved similarly high concordance (over 95%) when compared with manual abstraction [17].

These validation efforts serve to confirm the accuracy of the underlying extraction prompts and Versa GPT-4 API performance. The focus of the current work, therefore, is not on additional accuracy testing; rather, we build upon this foundation to present a modular, scalable implementation pipeline that operationalizes LLM-driven extraction at scale, within a secure, clinical-grade environment.

## Methods

### Overall Design

**Figure 1.**
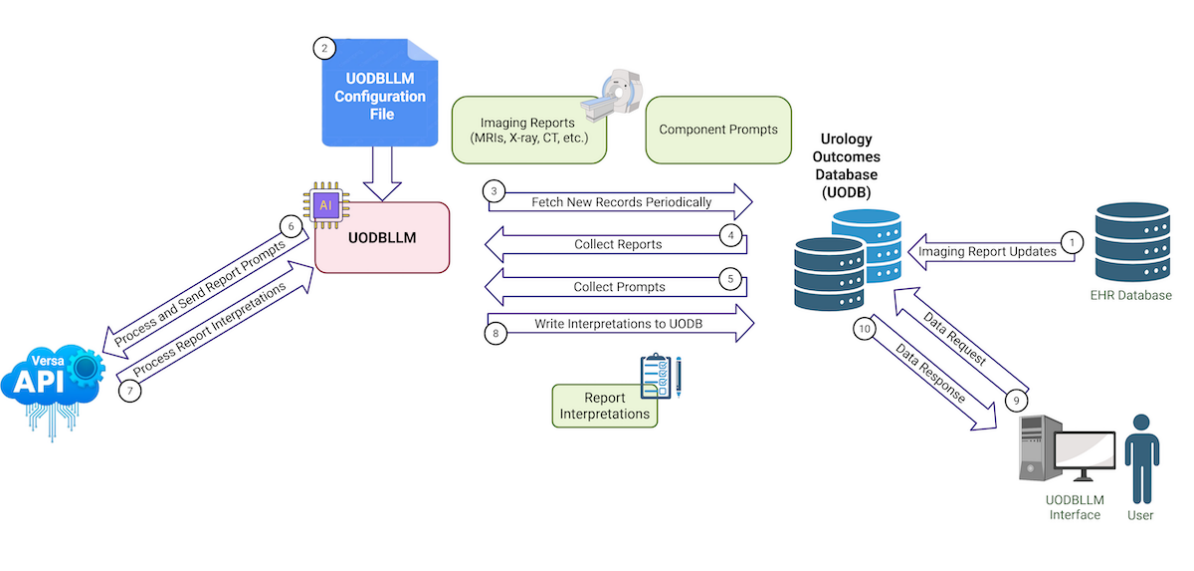
System design and data flow of the UODBLLM application. The process begins with an initial connection between the electronic health record (EHR) and the Urologic Outcomes Database (UODB) for imaging report updates (1). The UODBLLM application is governed by a configuration file defining its core parameters (2). The application periodically fetches new records from the UODB (3), collects the relevant reports (4) and component prompts (5), and sends these to the Versa application programming interface (API) for processing (6). The API returns structured interpretations of the reports (7), which are then written back into the UODB (8). A user, via the UODBLLM interface, can send a data request to the UODB (9) and receive a data response for review and analysis (10).

This study presents the implementation and performance evaluation of UODBLLM, a modular LLM-based pipeline designed for structured data extraction from a wide range of unstructured clinical reports. For this technical implementation, the system was evaluated using free-text prostate MRI radiology reports as the primary use case (Figure 1). The system was deployed within a secure, HIPAA-compliant clinical environment using the internal UCSF Versa GPT-4 API, ensuring that protected health information (PHI) remained confined to institutional systems. UODBLLM was designed with a flexible architecture to support multiple language models and API endpoints, enabling adaptability across varied clinical settings.

Prompts are stored as configurable components in dedicated database tables, allowing users to dynamically pair extraction templates with report sets without modifying the underlying code. This design supports rapid iteration, version control, and seamless adaptation to evolving information extraction needs.

### Study Population

The study dataset comprised 1800 prostate MRI radiology reports retrieved from the institutional EHR system. Reports were selected based on procedural coding and metadata filters to ensure relevance to downstream urologic data extraction.

### Intervention

UODBLLM is a Python-based (version 3.9.6, Python Software Foundation, worldwide) application designed to extract structured information from clinical reports using a modular, API-driven architecture. Source text is retrieved from the UODB using a parameterized SQL query passed via a secure Open Database Connectivity connection. Text blocks are staged and dispatched in configurable batches, controlled by a modifiable parameter specified in a configuration file or modifiable via command-line flag.

The pipeline retrieves a version-controlled extensible markup language (XML)-based prompt template at runtime using a parameterized SQL query from the UODB. This template specifies the role, task, JSON response schema, and a structured sub-prompt with 16 XML elements that each represent a clinical field of interest (eg, prostate volume, prostate-specific antigen density, and overall Prostate Imaging Reporting and Data System score), each with plain-language extraction instructions (Figure S1 in Multimedia Appendix 1). For every report, the program inserts the full free-text report into the template’s designated placeholder, producing a complete prompt that is then submitted to the Versa GPT-4 model. Embedding the report within a constant, schema-constrained envelope ensures that returned JSON follows a predictable structure, enabling reliable downstream parsing and storage.

Each batch is passed to a thin wrapper around the Versa GPT-4 API. Requests are streamed to the API endpoint; results are captured, parsed, and validated against the predefined JSON schema. Error handling includes up to 5 retry attempts per request with exponential back-off (2ⁿ seconds, capped at 30 seconds). Failed requests are logged, and the affected reports are re-queued for later processing. Element-level completeness is defined as the proportion of reports for which the pipeline returned a non-null value.

Extracted fields are transmitted back to the database using a set of parameterized SQL UPDATE statements mapped to internal column identifiers. A custom statistics tracking module records token usage, response latency, and processing cost per report by counting model-specific numerical tokens generated from text via Byte Pair Encoding. System-wide throughput and error frequency are also recorded. The pipeline was executed on a 2019 MacBook Pro (Intel Core i9, 2.4 GHz, 64 GB RAM, macOS Ventura 13.2.1). The system’s computational workload is lightweight and not hardware dependent, making it executable on a standard consumer laptop. The source code will be made available to investigators for non-commercial purposed upon request.

### Ethical Considerations

The study was approved by the University of California, San Francisco Institutional Review Board (IRB #11-05329), and the requirement for informed consent was waived. The system was deployed within a secure, HIPAA-compliant clinical environment using the internal UCSF Versa GPT-4 API, ensuring that PHI remained confined to institutional systems. All reports were de-identified prior to processing.

## Results

### Processing Performance and Resource Utilization

The analysis of system logs demonstrated consistent performance metrics, with an average processing speed of 8.90 seconds per report across 1800 reports. UODBLLM maintained 100% completion rates across all test runs, with batch sizes of 100 reports. Token utilization, representing the count of model-specific numerical tokens generated from the input and output text via Byte Pair Encoding (calculated using the tiktoken library), averaged 2692 tokens per report. Given the model’s context window capacity relative to typical report lengths, specific token optimization techniques like input text chunking were not required for this implementation. This resulted in an input-to-output ratio of approximately 13:2 (4,196,697 input tokens, 648,723 output tokens), resulting in an average processing cost of US $0.009 per report. The total processing run successfully analyzed all 1800 test reports in 4.45 hours, showing sustained performance at scale.

### Prior Validation

Although the present study did not re-evaluate extraction accuracy on this corpus, the underlying extraction logic and prompt structure have been previously validated in two independent studies by our group. In one effort involving 424 prostate MRI reports, the system achieved a median field-level accuracy of 98.1% (IQR 96.3%‐99.2%) for key clinical variables [21]. A subsequent study with 228 MRI reports demonstrated similarly high extraction fidelity, with all structured elements exceeding 95% accuracy [17]. These findings confirm the robustness of the prompt design and model configuration across settings, supporting their reliability in the context of the current implementation.

### Experience

Researchers interact with UODBLLM by selecting the clinical report category (eg, MRI reports or pathology reports) through a secure web-based application that integrates with the UODB and is accessible only through local institutional network connections. UODBLLM displays quantitative processing metrics for the selected report type, including extraction completion timestamps, LLM prompts, and performance statistics from previous analyses. This longitudinal view enables investigators to evaluate existing structured data’s temporal relevance and completeness before proceeding with additional processing.

Researchers can use previously extracted structured data or initiate a new extraction cycle with refined extraction parameters. When opting for new extraction, investigators can specify temporal bounds for report inclusion and modify extraction prompts stored in the database tables. This parameterization enables the analysis of specific clinical cohorts while ensuring consistent extraction methodology across research protocols.

Upon initiating the UODBLLM process, the system executes batch processing of identified reports, with real-time logging providing visibility into extraction progress. Researchers can monitor the system performance through logs that track processing times, success rates, and any encountered exceptions. The structured JSON output is automatically integrated into the UODB, enabling immediate access for researchers.

Quality assurance is implemented through a review interface where researchers can perform comparative analysis of extracted data elements against source reports and any pre-existing manually abstracted data with the opportunity to iteratively refine prompts. Successfully processed reports are flagged in the database, preventing duplicate processing while maintaining a comprehensive audit trail of all data extraction operations.

## Discussion

### Principal Findings and Comparison With Previous Works

In this study, we developed and validated an automated LLM-based integration for UODB management that achieved a 100% completion rate across 1800 clinical documents, with an average processing time of 8.90 seconds per report. The UODBLLM demonstrates an implementation of a PHI-secure, LLM-agnostic system for automated data extraction from urological outcomes documentation. By leveraging institutional cloud infrastructure and established database architecture, we created a scalable solution that significantly reduces the manual effort traditionally required for data extraction while maintaining high accuracy rates [19]. This advancement represents a crucial step toward efficient, accurate, and comprehensive research database management [18].

The integration of generative artificial intelligence in clinical data management has seen rapid evolution, with several institutions developing specialized approaches for extracting structured data from clinical documentation [1,2]. While the validation of a local GPT model showed promising accuracy in the low 90th percentile for biomedical data collection, their focus on chromatin expression in cell lines addressed a more constrained data domain [20]. UODBLLM demonstrates comparable accuracy rates with the ability for researcher customization. Recent oncology initiatives using LLMs for clinical note evaluation have shown potential, but our approach differs by providing a complete pipeline that not only extracts data but also integrates directly with existing database infrastructure [5,6]. The problem of integration from clinical care to research database is common in clinical trials, clinical record management, and safety reports, encouraging other groups to design automated data capture and transfer pipelines. These pipelines have historically been evaluated as successful by the variables they extract, efficiency gained, and interoperability they provide, aligning with our key performance indicators [22,23]. The pipeline here described and designed has been estimated to improve data extraction manual time efficiency by as much as 90% if pulling multiple variables from hundreds of reports, although this enhancement varies based on report type, variable, and iterations of prompt refinement.

The technical robustness of our approach is supported by key design decisions and validated through comprehensive testing. Our choice to leverage a PHI-secure institutional version of GPT-4 addresses performance and privacy requirements, crucial considerations for clinical data management [5]. The system’s integration within the UODB piggybacks off a validated foundation for data structure and management [19]. Our validation protocol included processing reports across various batch sizes, achieving consistent performance and reliable operation at scale. The ability of the UODBLLM to efficiently process clinical documentation while maintaining high accuracy suggests the potential for significant resource optimization in research operations [6]. These efficiency gains could dramatically accelerate research timelines and expand the scope of feasible clinical studies.

Although this study did not re-assess extraction accuracy, this was a deliberate design choice. The extraction framework employed here has already undergone validation in prior work, with element-level accuracies exceeding 95% across multiple prostate MRI cohorts [17,21]. In contrast, our current objective was to evaluate the system-level performance of a scalable, generalizable implementation pipeline deployed within a secure clinical environment. Notably, the architecture is model-agnostic and allows for future integration of various LLMs or prompt schemas. This decoupling of model validation from pipeline implementation facilitates adaptability while building on established, validated components.

The limitations of our approach warrant careful consideration. While UODBLLM performs robustly for current use cases, the accuracy of LLM-based data extraction still requires human validation for critical data points, a challenge noted across multiple studies [4,5,8]. The evolving nature of clinical research means that prompt engineering must continually adapt to new data types and research questions. Additionally, while our pipeline is LLM-agnostic, our specific performance results were achieved using a PHI-secure version of GPT-4, and performance may vary with different models or implementations. While this implementation focused on prostate MRI reports, the UODBLLM pipeline was designed for broad applicability across diverse clinical documents. This generalizability is enabled by its modular, model-agnostic architecture and a flexible prompting system where extraction templates are stored as configurable components in the database. The design allows the pipeline to be readily adapted for other unstructured texts, such as pathology results or operative notes, which aligns with plans to expand its use to other urologic cancers.

### Conclusions

Our study demonstrates the feasibility and effectiveness of integrating LLM-based automation into UODB management. Our system’s perfect completion rate, rapid processing speed, and cost-effective operation provides a robust framework for modernizing clinical research data management. Looking ahead, we aim to develop protocols for using LLMs to validate existing data entries and expanding to renal and bladder cancer radiology and pathology texts. The potential benefits of increased research efficiency and data quality suggest that LLM-based approaches will play an increasingly important role in clinical research infrastructure [4]. These advances may ultimately accelerate the pace of discovery in clinical oncology and serve as a model for other medical specialties.

## Supplementary material

10.2196/70708 Multimedia Appendix 1 Example of the UODBLLM Data Extraction Workflow. (A) The original unstructured text from a sample magnetic resonance imaging report. (B) The corresponding extensible markup language-structured prompt containing instructions and specific data extraction queries sent to the large language model (LLM). (C) The structured JSON data returned by the LLM based on the prompt and report.

## Abbreviations

API: application programming interface
EHR: electronic health record
GPT: generative pre-trained transformer
HIPAA: Health Insurance Portability and Accountability Act
LLM: large language model
MRI: magnetic resonance imaging
UCSF: University of California, San Francisco
UODB: Urologic Outcomes Database
XML: extensible markup language
