# Supplementary material for: Development and Validation of a Generative Artificial Intelligence-Based Pipeline for Automated Clinical Data Extraction From Electronic Health Records: Technical Implementation Study
Source: JMIR Bioinform Biotechnol. 2026 Jan 6;7:e70708. doi: 10.2196/70708 (PMC12774397; doi:10.2196/70708)
Supplement: Multimedia Appendix 1 [file bioinform-v7-e70708-s001.docx]

**Supplementary Figure S1:** Example of the UODBLLM Data Extraction Workflow. Panel A shows the original unstructured text from a sample MRI report. Panel B displays the corresponding XML-structured prompt containing instructions and specific data extraction queries sent to the LLM. Panel C presents the structured JSON data returned by the LLM based on the prompt and report.

| A. Original Unstructured Data  MR PROSTATE WITH AND WITHOUT CONTRAST 12/7/2024 3:56 PM COMPARISON: 12/14/2023 MR prostate CLINICAL HISTORY: 72 years man with elevated PSA, suspected prostate cancer , follow up of PI-RADS 2 lesion; Gleason score = N/A Management = active surveillance Most recent PSA = 7.8 ng/ml; PSA date = 01/2024 TECHNIQUE: Multiplanar T1, T2, and diffusion weighted MR images were obtained through the pelvis on a 3 Tesla magnet. Dynamic post-gadolinium images were acquired following the intravenous administration of gadolinium. 3D post-processing and segmentation of the prostate was performed in an independent workstation (DynaCAD) in preparation for possible MRI-ultrasound fusion biopsy with UroNav. MEDICATIONS: Dotarem - 16 mL - Intravenous FINDINGS: Prostate volume: 118 cc PSA density: 0.07 ng/ml2 Length of membranous urethra: 15 mm Multiparametric MR evaluation: Heterogeneous appearance of the central gland is consistent with benign prostatic hyperplasia. Lesion 1: Left mid medial peripheral zone; 0.9 x 0.6 cm; series 5 image 20 No suspicious lesions seen on T2-weighted MR imaging (T2 score = 2/5). No suspicious findings seen on diffusion-weighted MR imaging (DWI score = 2/5). ADC value: 1382 No suspicious enhancement identified (DCE negative). Overall PI-RADS v2.1 score = 2 Capsular margin and neurovascular bundle: No evidence of macroscopic extracapsular extension. Seminal vesicles: No evidence of seminal vesicle invasion. Lymph nodes: No lymphadenopathy seen in the field of view. Bones: No suspicious lesions in the field of view. Bladder: Trabeculated without focal lesions. Rectum/colon: Scattered colonic diverticuli without evidence of acute diverticulitis. Other: None IMPRESSION: - PI-RADS v2.1 score 2: clinically significant cancer is unlikely to be present. - No evidence of macroscopic extracapsular extension. No evidence of seminal vesicle invasion. - No lymphadenopathy. No suspicious bone lesions. - No significant changes seen since the prior MRI. |
| --- |
| B. LLM Prompt Structure  <report>  [insert report from section A.]  </report>  <instructions>  <role>You are an expert medical imaging report reader.</role>  <task>  Assume the <role>, and for the <report>, respond to each <prompts><prompt_object><prompt>. Use <response_format> to format the response.  </task>  <response_format>  A JSON array that follows the schema in <json_schema>. For the "Element" object, use the <prompts><prompt_object><data_element> value. Store the response in the "Value" field.  </response_format>  <json_schema>  {  "type": "array",  "items": {  "type": "object",  "properties": {  "Element": {  "type": "string"  },  "Value": {  "type": "string"  }  },  "required": ["Element", "Value"],  "additionalProperties": false  }  }  </json_schema>  <prompts>  <prompt_object>  <data_element>prostate_volume</data_element>  <prompt>Tell me what the prostate volume in cubic centimeters (cc) is in this report. Sometimes the prosate volume is reported in grams (g) or milliliters (mL), but both of these should be converted to cc with 1 mL = 1 cc and 1 g = 1 cc. If you cannot find the data, return "NA". Do not include units with values, and make all letters lowercase.</prompt>  </prompt_object>  <prompt_object>  <data_element>most_recent_psa</data_element>  <prompt>Tell me the most recent PSA (prostate specific antigen) measurement in this report. If you cannot find the data, return "NA". Do not include units with values, and make all letters lowercase.</prompt>  </prompt_object>  <prompt_object>  <data_element>psa_density</data_element>  <prompt>Tell me what the PSA (prostate specific antigen) density is in this report. If the PSA density is not listed in the report, calculate by finding the most recent PSA and divide that by prostate volume. If you cannot find the data, return "NA". Do not include units with values, and make all letters lowercase.</prompt>  </prompt_object>  <prompt_object>  <data_element>length_of_membranous_urethra</data_element>  <prompt>Tell me what the length of the membranous urethra in millimeters is in this report. If you cannot find the data, return "NA". Do not include units with values, and make all letters lowercase.</prompt>  </prompt_object>  <prompt_object>  <data_element>number_of_possible_lesions</data_element>  <prompt>Tell me what the number of possible lesions is in this report. Sometimes lesions are listed as dimenstions with descriptions; for example "a 10 x 8mm area of restricted diffusion" represents a lesion. If you cannot find any suspicious lesions, return 0. Do not include units with values, and make all letters lowercase.</prompt>  </prompt_object>  <prompt_object>  <data_element>overall_pirads_score</data_element>  <prompt>Tell me what is the PI-RADS Score for each lesion in this report as a list of numbers. For example, three lesions with PIRADS scores of 5, 4, and 4 should return "[5, 4, 4]". If there are both v1 and v2 PI-RADS scores reported, return the v2 scores. If you cannot find the data, return "NA" in the field. Do not include units with values, and make all letters lowercase.</prompt>  </prompt_object>  <prompt_object>  <data_element>extracapsular_extension</data_element>  <prompt>Tell me whether there is extracapsular extension confirmed or suspected in this report. Do not count microcapsular involvment as extracapsular extenstion. You may only answer "1" for "yes" or "0" for "no". If you cannot find the data, return 0. Do not include units with values, and make all letters lowercase.</prompt>  </prompt_object>  <prompt_object>  <data_element>seminal_vesicle_involvement</data_element>  <prompt>Tell me whether there is seminal vesicle involvement confirmed or suspected in this report. You may only answer "1" for "yes" or "0" for "no". If you cannot find the data, return 0. Do not include units with values, and make all letters lowercase.</prompt>  </prompt_object>  <prompt_object>  <data_element>enlarged_lymph_nodes</data_element>  <prompt>Tell me whether are there any enlarged lymph nodes found in this report (lymph nodes are definitely enlarged if greater than 0.8cm). You may only answer "1" for "yes" or "0" for "no". If no lymph node enlargement or lymphadenopathy is noted, return 0. Do not include units with values, and make all letters lowercase.</prompt>  </prompt_object>  <prompt_object>  <data_element>bone_lesions</data_element>  <prompt>Tell me whether there are bone lesions seen in this report. Bone islands do not count as bone lesions. You may only answer "1" for "yes" or "0" for "no". If you cannot find the data, return "NA". Do not include units with values, and make all letters lowercase.</prompt>  </prompt_object>  <prompt_object>  <data_element>clinical_cancer_stage</data_element>  <prompt>Tell me what is the clinical cancer stage in this report. If you cannot find the data, return "NA". Do not include units with values, and make all letters lowercase.</prompt>  </prompt_object>  <prompt_object>  <data_element>lesion_left_side</data_element>  <prompt>Is there a lesion or cancer present in the left side of the prostate? Return your answer as "1" for "yes" or "0" for "no". If you cannot find the data, return "NA". Do not include units with values, and make all letters lowercase.</prompt>  </prompt_object>  <prompt_object>  <data_element>lesion_right_side</data_element>  <prompt>Is there a lesion or cancer present in the right side of the prostate? Return your answer as "1" for "yes" or "0" for "no". If you cannot find the data, return "NA". Do not include units with values, and make all letters lowercase.</prompt>  </prompt_object>  <prompt_object>  <data_element>lesion_in_apex</data_element>  <prompt>Is there a lesion or cancer present in the apex of the prostate? Return your answer as "1" for "yes" or "0" for "no". If you cannot find the data, return "NA". Do not include units with values, and make all letters lowercase.</prompt>  </prompt_object>  <prompt_object>  <data_element>lesion_in_mid</data_element>  <prompt>Is there a lesion or cancer present in the middle or mid of the prostate? Return your answer as "1" for "yes" or "0" for "no". If you cannot find the data, return "NA". Do not include units with values, and make all letters lowercase.</prompt>  </prompt_object>  <prompt_object>  <data_element>lesion_in_base</data_element>  <prompt>Is there a lesion or cancer present in the base of the prostate? Return your answer as "1" for "yes" or "0" for "no". If you cannot find the data, return "NA". Do not include units with values, and make all letters lowercase.</prompt>  </prompt_object>  </prompts>  </instructions> |
| C. Generated Structure Output  [  {  "proc_id": "615370363",  "response": [  {  "Element": "prostate_volume",  "Value": "118"  },  {  "Element": "most_recent_psa",  "Value": "7.8"  },  {  "Element": "psa_density",  "Value": "0.07"  },  {  "Element": "length_of_membranous_urethra",  "Value": "15"  },  {  "Element": "number_of_possible_lesions",  "Value": "1"  },  {  "Element": "overall_pirads_score",  "Value": "[2]"  },  {  "Element": "extracapsular_extension",  "Value": "0"  },  {  "Element": "seminal_vesicle_involvement",  "Value": "0"  },  {  "Element": "enlarged_lymph_nodes",  "Value": "0"  },  {  "Element": "bone_lesions",  "Value": "0"  },  {  "Element": "clinical_cancer_stage",  "Value": "na"  },  {  "Element": "lesion_left_side",  "Value": "1"  },  {  "Element": "lesion_right_side",  "Value": "0"  },  {  "Element": "lesion_in_apex",  "Value": "0"  },  {  "Element": "lesion_in_mid",  "Value": "1"  },  {  "Element": "lesion_in_base",  "Value": "0"  }  ]  }  ] |
